# A Phenome-Wide Comparative Analysis of Individualized Network Heterogeneity Across Treatment-Response Subphenotypes in Coronary Heart Disease

**DOI:** 10.3390/biology15110843

**Published:** 2026-05-28

**Authors:** Shuang Guan, Yinli Shi, Sicun Wang, Yuanyuan Leng, Yanan Yu, Jun Liu, Zhong Wang

**Affiliations:** Institute of Basic Research in Clinical Medicine, China Academy of Chinese Medical Sciences, Beijing 100700, China; shuangg46@163.com (S.G.);

**Keywords:** heterogeneity of treatment effect, group-level, individual-level, network analysis, individualized network

## Abstract

To address the heterogeneity in treatment effects (HTE) in precision medicine for coronary heart disease (CHD), we employed an individualized network analysis framework (Pheno-NM) to elucidate the molecular mechanisms of HTE in patients treated with Danhong injection (DHI). We integrated clinical phenotyping and transcriptomic data to identify three efficacy-based subgroups. The best-responding subgroup (D(+)S(+)) displayed the most complex gene network, with its key hub gene ORM1 linked to platelet activation. Individualized network analysis revealed that patient-specific symptom improvement correlated with unique functional module connectivity and gene expression variations (e.g., HSBP1L1 and KCNG2). Furthermore, six core network topological parameters significantly correlated with treatment efficacy and differed between subgroups (*p* < 0.05), alongside significant differential expression of genes such as IQCD and MTFR1. This work establishes a novel joint phenotype-genetic network modeling paradigm, providing a molecular framework for HTE and paving the way for precise, personalized cardiovascular interventions by revealing patient-specific network architectures.

## Simple Summary

Coronary heart disease patients often respond differently to the same treatment, making personalized care difficult. This study assessed a widely used herbal injection for heart disease to elucidate the variations in treatment effects observed between people. We used patient symptoms and transcriptome data to split patients into three groups based on how well they improved. The best-responding cohort demonstrated the most intricate gene-network architecture. This study also observed that each patient’s unique gene network pattern matched their own symptom changes. These findings help explain why some patients benefit more from this treatment, providing doctors with a method to select patients for better outcomes. This work supports more precise, personalized care for coronary heart disease and helps improve treatment safety and efficacy for more people.

## 1. Introduction

Recognizing and addressing the heterogeneity in treatment effects (HTE) represents a significant challenge in the advancement of precision medicine [1,2]. While the identification of optimal therapies tailored to individual patients holds transformative potential for both individual and population health, current evidence-based paradigms often fail to capture the complexities inherent in patient-specific treatment responses [3]. While randomized clinical trials (RCTs) typically report average treatment effects, these aggregate results fail to account for the variability in individual responses observed in clinical practice, revealing a critical gap in understanding treatment efficacy at the individual level [4]. This divergence underscores the central paradox of modern clinical research: although RCT-derived average effects provide essential evidence for regulatory decisions and guideline development, they often fail to capture the biological and clinical heterogeneity that determines real-world treatment effectiveness [5]. Despite the widespread use of subgroup analyses, existing analytical methods remain limited in their ability to detect HTE due to challenges such as multiple comparisons, inadequate statistical power, and potential spurious associations. To overcome these limitations, emerging methodologies such as machine learning-based subphenotyping, counterfactual outcome prediction models, and Bayesian hierarchical effect estimation frameworks offer promising solutions that enable precise detection [6,7]. However, significant barriers remain in translating these analytical advances into practice, particularly in validating predictive biomarkers and developing complex prediction models in clinical workflows.

Precision medicine is centered around three critical questions: which therapies are most effective, which patients are most likely to benefit, and in which biological contexts do these treatments perform best? [8] This paradigm shift from population-based to molecularly guided care is exemplified by the successful implementation of genotype-matched therapies in oncology, where tumor genomic profiling directs targeted treatment selection [9]. The success of genotype-matched therapies in oncology has been a key driver for adopting molecularly guided treatment paradigms, and similar approaches are now being explored for chronic diseases, such as stable angina and type 2 diabetes, where genetic and molecular variations contribute to differential therapeutic responses. Building on these insights, our study utilizes phenotype-based clustering and network-driven analysis to uncover the molecular mechanisms underlying HTE in cardiovascular and metabolic diseases [10,11,12].

**Figure 1:**
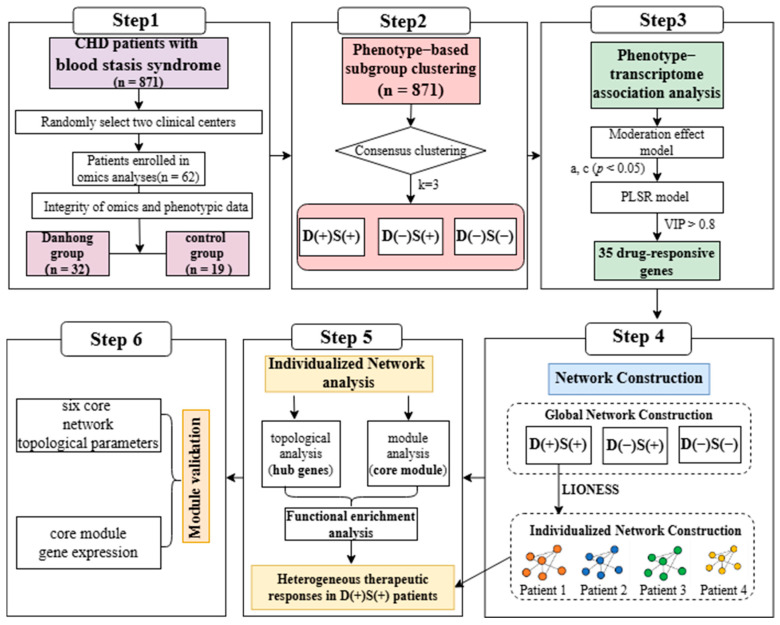
Workflow of the Individualized Network Analysis Framework (Pheno-NM).

Danhong injection is a multi-component traditional Chinese medicine preparation extracted from Danshen and Honghua. Its principal constituents include sodium danshensu, protocatechuic aldehyde, p-coumaric acid, rosmarinic acid, and salvianolic acid [13]. DHI has been reported to promote endothelial progenitor cell mobilization by upregulating Akt, eNOS, and MMP-9 expression; attenuating atherosclerosis and macrophage lipid accumulation through activation of the PI3K/Akt insulin signaling pathway; reducing platelet aggregation; and mitigating oxidative stress while preserving mitochondrial integrity via the Keap1/Nrf2/JNK pathway [14,15,16,17], resulting in its wide use in China for cardiovascular indications. Furthermore, a number of clinical studies also demonstrated that DHI might be an effective and safe treatment option for preventing angina attacks in CAD management [18]. This study aims to enhance our understanding of the molecular pathways driving HTE while addressing the challenges of integrating predictive biomarkers and complex models into clinical workflows, thus paving the way for more personalized therapeutic strategies in clinical practice (Figure 1).

## 2. Methods

### 2.1. Study Design

This study was a post hoc exploratory analysis based on data from a completed adaptive-design, randomized, multicenter, double-blind, placebo-controlled trial. The trial protocol was approved by the central institutional review board of the Chinese PLA General Hospital (IRB No. [2012] Pharmaceutical (025)). This trial complied with the principles of the Declaration of Helsinki. Informed written consent was obtained from all participants. Patients or the public were not involved in the design, conduct, reporting, or dissemination plans of our research [19].

### 2.2. Participants

Symptomatic patients aged 18 to 70 years, diagnosed with coronary heart disease (CHD) and blood stasis syndrome (BSS, defined as a score of at least 15 on the Chinese Medicine Symptom Scale for angina patients), and classified with CCS class II or III angina, were eligible for enrollment. Key exclusion criteria included a history of myocardial infarction within the past 3 months, severe complications such as liver or renal dysfunction, severe cardiopulmonary dysfunction, and conditions like epilepsy or cerebral hemorrhage. Patients with a history of drug-induced bleeding, hematopoietic disorders, or surgery within the past 4 weeks were also excluded.

### 2.3. Selection Criteria for the Omics Analyses

Serum samples from patients at 301 Hospital and Xuanwu Hospital were collected and sequenced using the Illumina HiSeq platform, in accordance with the manufacturer’s instructions and established protocols. A total of 62 patients were enrolled in the sequencing cohort, including 41 cases in the Danhong injection group and 21 cases in the control group. After rigorous screening based on complete transcriptomic and phenotypic data availability before and after treatment, 51 coronary heart disease patients presenting with blood stasis syndrome were finally included in the omics analysis, with 32 patients in the Danhong injection group and 19 in the control group (Supplementary Table S1).

### 2.4. Phenotype-Based Subgroup Assignment

To identify phenotype-driven subgroups, consensus clustering was performed based on the blood stasis syndrome cluster (BSSC) and AF scores using the “ConsensusClusterPlus” package [20]. The K-means clustering algorithm was employed to determine the optimal number of clusters (k) by evaluating group numbers ranging from k = 2 to k = 10, and the number of clusters was determined by selecting the k value that maximized inter-group consistency, minimized the coefficient of variation, and exhibited a smooth cumulative distribution function (CDF) curve. To evaluate subgroup replicability, we employed an intra-center validation strategy across independent centers: First, we randomly selected two centers from the original dataset (comprising 20 centers) as an independent validation set, with the remaining data serving as the training set. Using consensus clustering, we determined the optimal subgroup count within the training set and calculated the centroid features for each subgroup. Subsequently, samples from the validation set were assigned to the nearest subgroup centroid in the training set to verify subgroup reproducibility across independent datasets.

### 2.5. Phenotypes-Driven Correlation with Transcriptomes

In this study, we hypothesized that drug response is influenced by baseline pathological levels, and a baseline phenotype space was constructed, with the BSSC score on the horizontal axis and the AF score on the vertical axis, to visualize the spatial patterns of clinical phenotypes.

A moderation effect model was used with the PROCESS() function from the bruceR package in RStudio (4.4.0), with the AFD30 value as the dependent variable (*Y*), gene expression levels as the independent variable (*X*), and baseline AFD0 and BSSCD0 as moderators (*M*1, *M*2), and its formula is as follows:

(1) Y=a⋅X+b⋅Z+c⋅X⋅Z+ε

where *a*, *b*, and *c* are regression coefficients, *X* represents gene expression, *Z* represents moderators (*M*1, *M*2), (*X·Z*) is the interaction term, and *ε* is the error item. In this study, we selected genes with statistically significant regression coefficients *a* and *c* for subsequent analysis. The Benjamini–Hochberg method was used to adjust for multiple testing, and both raw *p*-values and adjusted *p*-values (*p*-adj values) were reported.

### 2.6. Phenotype-Gene Correlation Analysis

We established an association model between genes and the clinical phenotypes (AF and BSSC). Here, absolute changes in gene expression were used as independent variables, while changes in AF and BSSC scores served as dependent variables. Partial Least Squares Regression (PLSR) was conducted using the pls package in RStudio (4.4.0) to identify drug-responsive genes, with Variable Importance in Projection (VIP) set as the selection criterion, at a threshold of VIP > 0.8 [21]. The correlation between genes and clinical phenotypes was assessed using Spearman’s correlation, and the relationship between core modules and phenotypes was analyzed using canonical correlation analysis (CCA).

### 2.7. Global and Individualized Network Construction

#### 2.7.1. Global Network Construction

We selected drug-responsive genes as nodes and constructed a co-expression network using Pearson correlation analysis for each time point (Day0 and Day30), and edges with a correlation coefficient change greater than 0.3 between the two time points were retained.

#### 2.7.2. Individualized Network Construction

Individualized networks were constructed using the LIONESS method [22]. This approach estimates an individualized network by integrating information from both an aggregate network and one excluding the individual of interest, thereby assessing how the removal of this sample impacts the network.

### 2.8. Network Structure Analysis

The CytoHubba plugin for Cytoscape (3.10.3) was applied to both global and individualized networks to identify hub genes, and node importance was evaluated using three centrality measures: Degree, Closeness Centrality, and Betweenness Centrality [23].

### 2.9. Modular Map Construction and Intermodule Connection Quantification

Individualized networks were clustered by GN-weight to detect structural modules based on network edge weights. To understand the coarse architecture of the individualized networks, a modular map was constructed where each vertex represented a module, and edge width was proportional to intermodular connection strength. The strength of direct and indirect inter-module connections was quantified using the SW method [24].

### 2.10. Core Module Identification of the Modular Map

To identify core modules contributing to inter-module information propagation, we ranked the modules by their average degree in the modular map, and the top-ranked module was defined as the core module.

### 2.11. GO Biological Processes and KEGG Signaling Analysis

Gene Ontology (GO) biological process and KEGG pathway enrichment analyses were performed for each module to identify regulatory functions and pathways. Metascape [25] (https://metascape.org) was then used for enrichment analysis, with a significance threshold set at *p*-adj < 0.05.

### 2.12. Genes Module Verification

To validate the association between the core module and BSSC symptoms, we performed a text mining analysis to determine if the top genes in the core module were linked to behavioral patterns observed in three patients. The stability of the core module’s structure was validated using six network topology parameters (subnetwork weighted edge, internal density, average internal degree, cut size, conductance and modularity contribution), thereby confirming that the identified module structure remained stable across different efficacy subgroups and was conserved in distinct patient subgroups. Additionally, inter-group differences were analyzed using the independent samples *t*-test.

## 3. Results

### 3.1. Clustering Identifies Subgroups of Coronary Heart Disease Based on Phenotypic Characteristics

**Figure 2:**
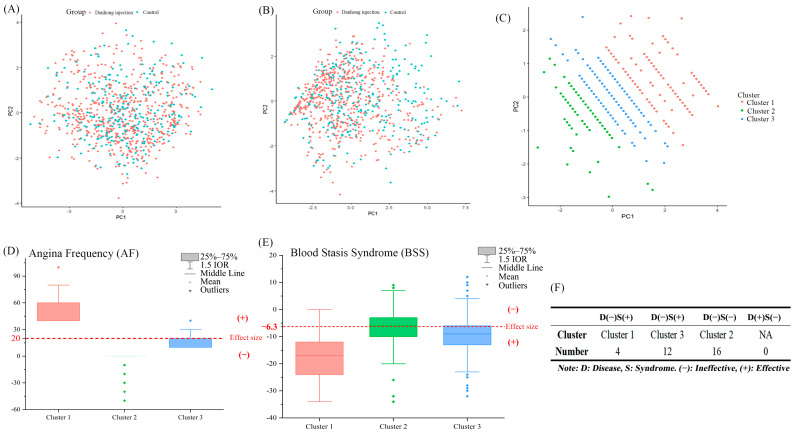
Changes in Clinical Phenotypes Define Three Coronary Heart Disease Blood Stasis Syndrome Efficacy Subgroups. (**A**,**B**) show the distribution of patient characteristics (N = 871), (**A**) before treatment and (**B**) after 30 days of treatment. (**C**) shows the consistency clustering results for all patients. (**D**,**E**) display disease and symptom improvements for patients in three categories. (**F**) shows the subgrouping results for the therapeutic effect of Danhong injection on patients.

A total of 871 patients participated in the study, with 582 and 289 assigned to the treatment (66.8%) and control groups (33.2%), respectively. The mean age of the overall cohort was 59.54 years, with 64.52% of participants being male, and there were no significant differences in baseline characteristics between the two groups. Figure 2 provides a visual representation of the phenotypic characteristics of all participants. Prior to treatment (Day 0), patients in the treatment group exhibited a uniform distribution of phenotypic features. However, after 30 days of treatment (Day 30), a more clustered distribution of these characteristics was observed. In contrast, the phenotypic distribution of patients in the control group showed no significant changes before and after treatment (Figure 2A,B), with clustering analysis conducted to determine whether CHD and BSS patients naturally form homogeneous subgroups based on the two spatial dimensions: disease (D) and BSSC (S). According to the CDF curve, the consensus clustering along these dimensions identified k = 3 as the optimal number of subgroups (Figure 2C and Supplementary Figure S1A,B), highlighting the heterogeneity within the treatment group based on phenotypic characteristics.

Subsequent analysis identified three subgroups based on phenotype improvement (efficacy). Specifically, clinical response D(+) was defined as an improvement of more than 20 points in AF value from the baseline, and syndrome response S(+) was defined as a reduction of over 30% in syndrome score from the baseline. The clinical indication of the subgroup labels were based on the patients’ disease status and BSS syndrome phenotypic improvement: D refers to disease response, while S refers to BSS syndrome response; “+” indicates efficacy reaching the prespecified evaluation standard, while “−” suggests no effective improvement, and therefore, the populations were divided into three categories: (1) Category1: D(+)S(+) referred to the best drug-responsive subgroup; (2) Category2: D(−)S(+) referred to only the symptoms-improvement subgroup; and (3) Category3: D(−)S(−) indicated the poor drug-responsive subgroup (Figure 2D–F and Supplementary Table S2).

Based on the three subgroup centers obtained from the training set, we employed the nearest center method to assign independent validation set samples to their respective subgroups. The results demonstrated that the three subgroup structures were well reproduced in the validation set, with sample proportions highly consistent with those in the training set and stable phenotypic characteristics and inter-group differentiation patterns across subgroups, confirming the good reproducibility and stability of the identified subgroups (Supplementary Figure S1C,D).

### 3.2. Transcriptomic Features of DHI Treatment Response

**Figure 3:**
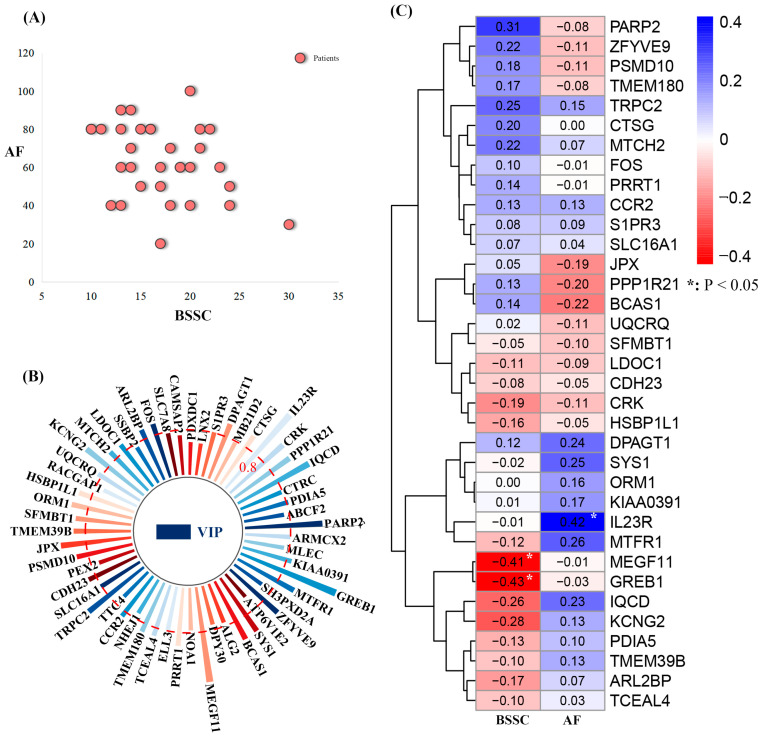
Analysis of Transcriptome and Clinical Phenotype. (**A**) Space schematic diagram of baseline disease in patients. (**B**) Drug-responsive genes, and (**C**) drug-responsive genes and phenotype correlation (*: *p* < 0.05).

Previous studies have highlighted the significance of network analysis in understanding the mechanisms behind DHI’s efficacy in treating CHD [19]. To further investigate this, we constructed a baseline phenotype space for CHD patients treated with DHI using baseline phenotypic data (Figure 3A).

We then compared patients in the Danhong injection group with those in the control group and identified 55 candidate genes (*p* < 0.05) that were potentially associated with treatment response (Supplementary Table S3). To examine the relationship between these genes and clinical phenotype changes (treatment effects), Partial Least Squares Regression (PLSR) was applied. This analysis identified 35 drug-responsive genes (VIP > 0.8) that were significantly associated with the treatment effects of DHI (Figure 3B), and further analysis revealed that three genes, MEGF11, GREB1, and PARP2, showed significant correlations with changes in BSSC (r > 0.3). Among these, GREB1 was the most correlated with BSSC (r = −0.43), and IL23R showed the highest correlation with AF (r = 0.42) (Figure 3C). These findings suggest that DHI’s efficacy is influenced by multiple genes, rather than a single gene, indicating a complex underlying mechanism.

### 3.3. Dynamic Gene Network Connectivity in Subgroups

Next, we hypothesized that DHI modulates the pathophysiology of CHD patients by influencing dynamic gene network connectivity. Gene co-expression network analysis from each subgroup revealed distinct sets of genes relevant to each subgroup, highlighting the varying gene networks that contribute to treatment response. Further examination of network nodes showed that the D(+)S(+) and D(−)S(+) subgroups contained all efficacy-related genes, while the D(−)S(−) subgroup accounted for 62.9% of all drug-responsive genes. Network analysis also identified hub genes for each subgroup: CCR2, TMEM180, TRPC2, GREB1, FOS, IQCD, TMEM39B, and ORM1 in the D(+)S(+) subgroup, MTFR1, KIAA0391, KCNG2, SYS1, CCR2, BCAS1, PRRT1, and CTSG in the D(−)S(+) subgroup, and FOS, MEGF11, CCR2, SLC16A1, BCAS1, PRRT1, JPX, and PSMD10 in the D(−)S(−) subgroup. Importantly, CCR2 was identified as hub gene common to all three subgroups.

**Figure 4:**
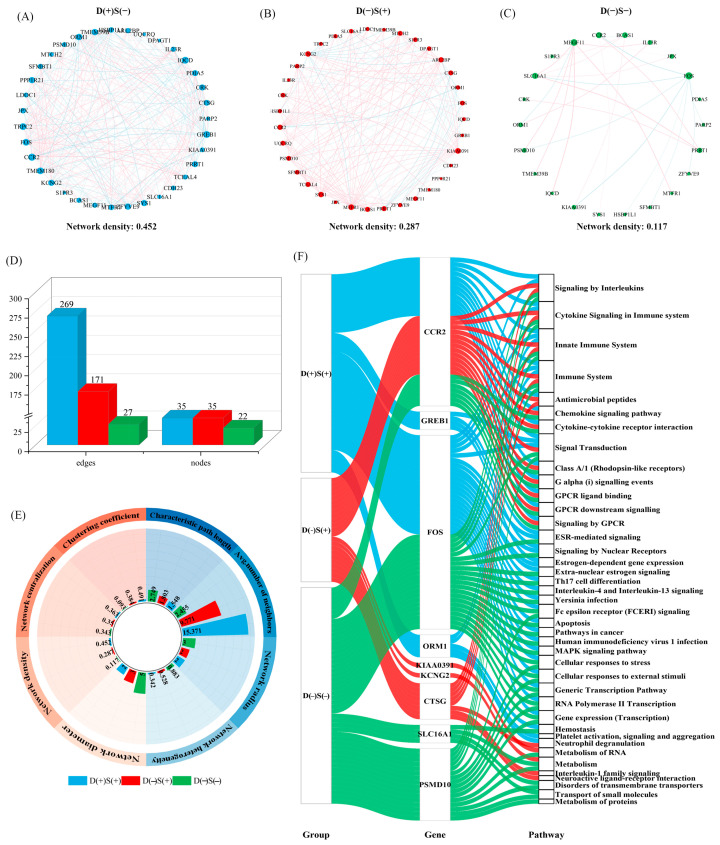
Network Analysis Reveals the Specificity of Each Efficacy Subgroup. (**A**–**C**) Gene networks of the three efficacy subgroups. (**D**,**E**) Network comparison analysis of the three efficacy subgroups. (**D**) Comparison between network edges and nodes of the three efficacy subgroups. (**E**) Comparison between network topological parameters of the three efficacy subgroups. (**F**) Network molecular mechanisms of three efficacy subgroups.

The D(+)S(+) subgroup exhibited the most complex network structure, while the D(−)S(−) subgroup had the sparsest network (Figure 4A–C), suggesting a positive correlation between network complexity and treatment efficacy. Network structure analysis further supported these findings (Figure 4D), as hub genes from the three subgroups were involved in distinct pathways. Notably, the D(+)S(+) subgroup was associated with ORM1-related platelet activation, signaling, and aggregation (Figure 4E), highlighting a specific mechanism that contributes to the treatment efficacy in this subgroup.

### 3.4. Individualized Network Signatures in the D(+)S(+) Subgroup

**Figure 5:**
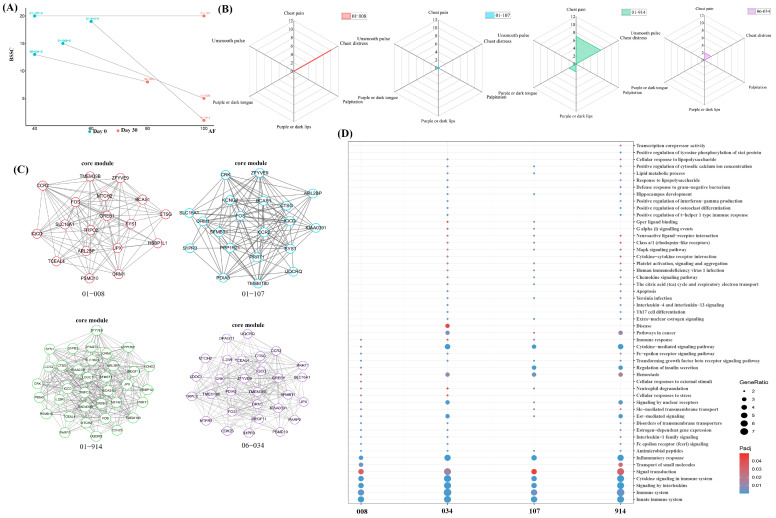
Heterogeneity of Therapeutic Effects in Patients with D(+)S(+). (**A**) Changes in the clinical phenotypes of diseases in D(+)S(+) subgroup patients. (**B**) Changes in the BSSC of D(+)S(+) subgroup patients. (**C**) Hub individualized network modules of D(+)S(+) subgroup patients. (**D**) Molecular mechanisms of D(+)S(+) subgroup patients.

In the previous analysis, we identified three subgroups (D(+)S(+), D(−)S(+), and D(−)S(−)) based on the efficacy of DHI treatment in CHD patients, with the D(+)S(+) subgroup showing the most complex network structure and the strongest treatment response. While some patients within this subgroup exhibit similar clinical phenotype changes, differences in their underlying physiological states and disease trajectories can lead to HTE. To explore this variability, we focused on individual patients within the D(+)S(+) subgroup for further network analysis. This subgroup consists of patients with satisfactory overall therapeutic responses, which eliminates confounding interference caused by poor general efficacy. Subsequently, we selected four patients with transcriptome data from this subgroup for individualized network analysis (Figure 5A).

Significant differences in BSSC clinical phenotypic changes were observed before and after treatment in four patients, with the most pronounced improvement seen in patient 914. Specifically, this patient showed improvements in chest pain, chest distress, purple or dark lips, and a purple or dark tongue. Patient 034 also showed improvements in chest pain and chest distress, but to a lesser degree compared to patient 914. Patient 008 only showed improvement in chest distress, while patient 107 did not show any improvement (Figure 5B).

To better understand these individualized responses, we constructed individualized networks for each of the four patients by maintaining identical nodes but varying edge weights based on their transcriptome data. The initial network analysis revealed differences in network structure between patients. However, to further explain how these structural differences relate to treatment efficacy, we proceeded with modular analysis. Modular analysis of these networks revealed distinct patterns of functional module connectivity, suggesting that each patient’s unique network structure contributes to the observed differences in treatment efficacy. This analysis also allowed us to identify core modules within each patient’s individualized network and conduct functional enrichment analysis of these modules, highlighting patient-specific variations in treatment response (Figure 5C,D).

### 3.5. Linear Changes in Disease Modules and Treatment Phenotypes Reveal HTE in CHD Patients

**Figure 6:**
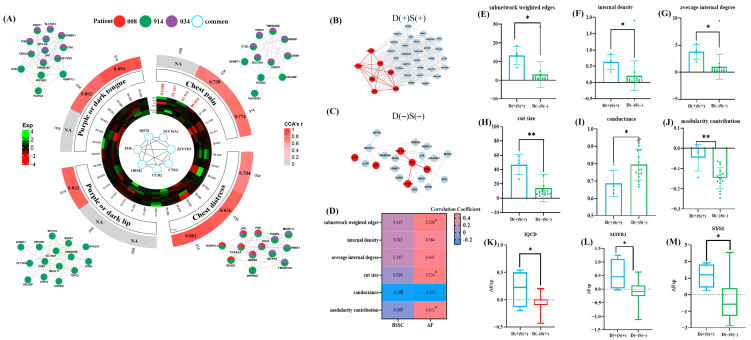
Phenotype-Driven Individualized Networks Module. (**A**) Network module associated with BSSC phenotypes (NA: no correlation). (**B**,**C**) Comparison of structure and network topology parameters of modules in different groups. (**D**) Correlation between six network topology parameters and clinical phenotypes (*: *p* < 0.05). (**E**–**J**) Six topological parameters of the common module differed significantly across efficacy subgroups. (**E**) subnetwork weighted edge (**F**) internal density (**G**) average internal degree (**H**) cut size (**I**) conductance (**J**) modularity contribution. (**K**–**M**) Expression level changes in IQCD, MTFR1 and SYS1 in different groups (*: *p* < 0.05; **: *p* < 0.01).

Changes in disease modules and treatment phenotypes during intervention were systematically evaluated, and correlations between core modules and clinical phenotypes in three patients showing improvement in BSSC were calculated, with correlation coefficients exceeding 0.7 (Figure 6A). These symptom modules were associated with distinct molecular pathways. Specifically, the chest pain module was linked to pathways involved in insulin secretion regulation and hemostasis, whereas the chest distress module was related to apoptosis.

Gene expression patterns within symptom modules varied among patients. HSBP1L1 and KCNG2 showed downregulation in patient 914 but were significantly upregulated in patient 034. In contrast, TMEM39B was downregulated in patients 008 and 034 but upregulated in patient 914. These findings reflect patient-specific variations in gene expression within the same symptom-related modules. Furthermore, a common module was identified across symptom modules, which may represent a core structure related to therapeutic response.

To further investigate the individualized mechanisms associated with BSSC, the structural integrity of this common module was validated. The D(+)S(+) subgroup exhibited tightly connected module structures, while the D(−)S(−) subgroup displayed sparser network connectivity (Figure 6B,C). Network topological parameters, including subnetwork weighted edge, internal density, average internal degree, cut size, conductance and modularity contribution, were calculated for this common module across different efficacy subgroups. Except for conductance, all parameters positively correlated with treatment phenotypes, and inter-group comparisons revealed significant differences in six network parameters (*p* < 0.05) (Figure 6D–L).

Additionally, these results were supported by comparative analysis of gene expression profiles across functional modules supported these results. Statistically significant differential expression of IQCD was observed between the D(+)S(+) and D(−)S(+) subgroups (*p* < 0.05), and MTFR1 expression also showed significant variation between the D(+)S(+) and D(−)S(−) subgroups (*p* < 0.05) (Figure 6G–M). These findings underscore the close association between individualized network module characteristics and clinical treatment outcomes.

## 4. Discussion

The network-based approach has allowed researchers to easily explore the pharmacological mechanisms of DHI for coronary heart disease treatment [26]. However, limited understanding of the drug’s heterogeneous efficacy has restricted the development of therapeutic intervention strategies [27]. In this study, a network-based individualized phenotyping technique was used to reveal the network molecular features of the HTE of DHI.

Our analysis identified a subgroup with a better therapeutic response, providing a new perspective for understanding the heterogeneous response to DHI. In this study, ORM1 was identified as the subgroup-specific gene in the best-efficacy group and was associated with platelet activation, signaling, and aggregation. This finding is consistent with previous reports suggesting that Danhong injection may exert anti-platelet effects through multi-target pathways, including reduced platelet aggregation and adhesion, as well as collagen metabolism regulation [28]. Orosomucoid 1 (ORM1), also known as alpha-1-acid glycoprotein, primarily functions as a transport protein in the bloodstream [29]. As an acute-phase protein and inflammatory factor, ORM1 has multiple biological activities [30], and it has been reported to stimulate the release of pro-inflammatory cytokines such as TNF, IL-1, IL-6, and IL-12 from monocytes, which may contribute to inflammatory responses [31,32]. Danhong injection is widely used in coronary heart disease treatment, and its pharmacological effects include promoting blood circulation, antithrombotic activity, anti-inflammatory effects, and myocardial protection [13].

**Figure 7:**
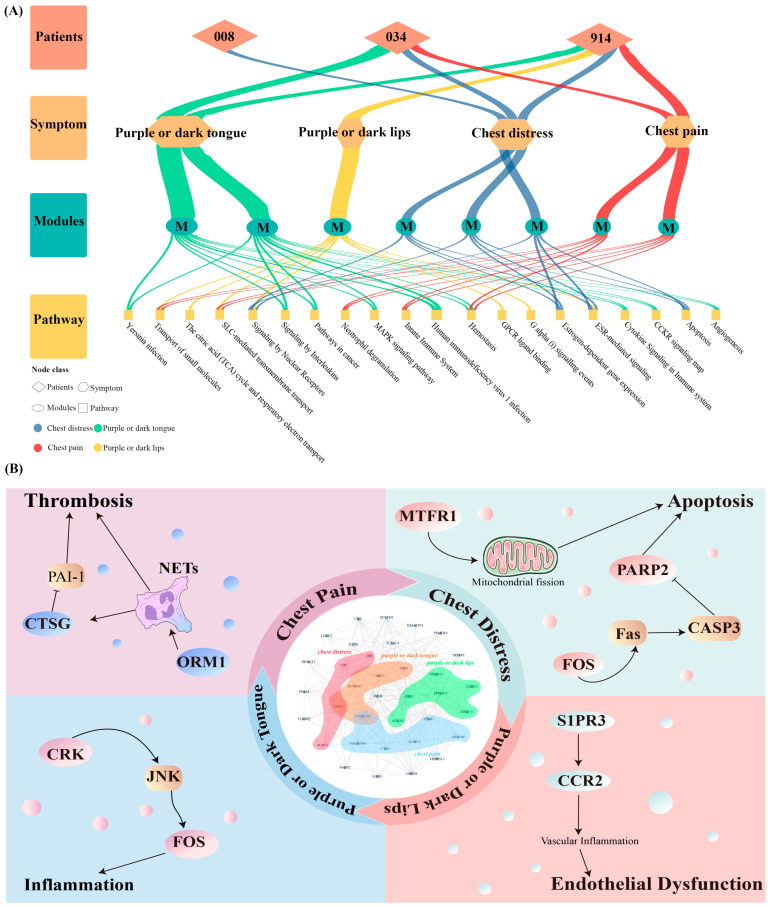
Functional Analysis of Symptom-Related Modules in Individualized Network Modules. (**A**) Pathway enrichment and biological function module analysis of individuals and symptoms in the D(+)S(+) subgroup. Lines are color-coded by BSSC types. (**B**) Molecular Mechanism of Danhong Injection in Treating BSSC.

However, treatment responses vary substantially among patients due to differences in genotype, pathophysiological characteristics, lifestyle, and environmental factors [33]. In this study, network analysis was used to explore these individualized responses, and a common module was identified. We found that this module was more tightly connected in the D(+)S(+) subgroup, suggesting a subgroup-specific network feature that may be associated with better efficacy. SYS1 has been implicated in GTPase synthesis by Arl3p [34]. Additionally, the GTPase activity regulation pathway involves three major classes of regulators in the Ras superfamily, namely GTPase-activating proteins (GAPs), guanine nucleotide exchange factors (GEFs), and guanine nucleotide dissociation inhibitors (GDIs). Previous studies have shown that members of the Ras superfamily, particularly Rho GTPases, are associated with the development of hypertension, heart disease, and diabetic nephropathy, and their regulators may influence vascular regulation through the Rho/ROCK pathway [35,36,37,38]. MTFR1, previously known as CHPPR (chondrocyte protein). It may regulate mitochondrial function and influence mitochondrial integrity. A previous study reported that miR-324-5p protects endothelial progenitor cells from oxidative stress-induced injury by regulating MTFR1 [39]. Under oxidative stress, glutaminolysis is upregulated to compensate for αKG depletion and support TCA cycle replenishment, which may exert cardioprotective effects by maintaining ATP and GSH levels [40] (Figure 7).

The traditional RCT aims to estimate the average effect of an intervention in the overall population by randomization to balance confounders, but it may not fully capture the influence of individual characteristics such as age, genotype, and comorbidities on treatment response [41]. In complex diseases or heterogeneous populations (e.g., diabetes and cancer), average effects may conceal patient-level differences and may not adequately reflect heterogeneity [42]. In this study, we identified disease-associated gene modules through gene network analysis and explored their patterns across patient subgroups. These results offer limited preliminary observations and may provide a modest basis for future research in personalized medicine. However, the present study has several limitations. First, the study was limited by the available dataset, which may affect the generalizability of the findings and highlights the need for validation in independent cohorts. Second, because the analyses were based on observational transcriptomic and network approaches, the biological interpretations should be considered preliminary and hypothesis-generating. Although the text mining analysis of key genes associated with the best-responding subgroup provided some supportive evidence, a direct link between subgroup-related genes and clinical phenotypes was not established. Future large-scale clinical studies with larger patient cohorts are therefore needed to further validate these findings.

## 5. Conclusions

Our study integrated clinical–transcriptional data, established a new paradigm of HTE analysis (joint phenotype–genetic network modeling) through individualized network analysis, and found the molecular network basis of DHI’s therapeutic heterogeneity. The results offer preliminary insights into possible molecular network features associated with this heterogeneity and may inform future studies on personalized treatment approaches.

## Data Availability

The data that support the findings of this study have been deposited in the CNSA (https://db.cngb.org/cnsa/) (accessed on 25 May 2026) of CNGBdb with accession number CNP0000461.

## Supplementary Materials

The following supporting information can be downloaded at: https://www.mdpi.com/article/10.3390/biology15110843/s1, Figure S1: Consensus clustering for subphenotype identification: optimal k selection and phenotypic validation; Table S1: Group of patients in Danhong injection group; Table S2: Clinical characteristics of three subgroups in the Danhong injection group; Table S3: Detailed information of the 55 candidate genes.
