# Supplementary material for: A Phenome-Wide Comparative Analysis of Individualized Network Heterogeneity Across Treatment-Response Subphenotypes in Coronary Heart Disease
Source: Biology (Basel). 2026 May 28;15(11):843. doi: 10.3390/biology15110843 (PMC13255898; doi:10.3390/biology15110843)
Supplement: Supplementary file 1 [file biology-15-00843-s001.zip › Supplementary Table.pdf]

Supplementary Table S1: Clinical characteristics of patients who provided serum samples for RNA sequencing

Characteristics	Total (N=871)	Total RNA sequencing populations (N=62)	RNA sequencing populations (N=51)	DHI group (N=32)	Control group (N= 19)
**Sex —No. (%)**					
Male	562	41	37	24	13
Female	309	21	14	8	6
**Age (yr) (Mean(Std))**	59.54(6.82)	58(6)	57.27(6.21)	57.50(5.59)	56.89(7.28)
**BMI(kg/m^2) (Mean(Std))**	24.67(2.89)	26.05(2.71)	26.04(2.71)	25.78(2.51)	26.49(3.03)
**SAQ domains (Mean(Std))**					
Physical limitation	67.17(12.38)	71(9)	71.80(10.05)	72.99(11.22)	69.82(7.57)
Angina frequency	56.93(22.02)	64(18)	65.49(17.70)	63.44(19.45)	68.95(14.10)
Treatment satisfaction	52.70(18.85)	66(12)	66.32(11.41)	65.81(13.15)	67.18(7.92)
Quality of life	38.57(18.51)	37(17)	38.07(16.44)	37.24(14.96)	39.47(19.01)
Angina stabilit	42.88(21.03)	42(22)	41.67(21.60)	41.41(23.43)	42.11(18.73)
**TCM syndrome score (Mean(Std))**	21.41(7.56)	16.77(4.52)	16.43(4.54)	17.16(4.60)	15.21(4.29)
**CCS angina class—No. (%)**					
II	689	54	46	28	18
III	182	8	5	4	1
**Plasma lipid level (mmol/L)(Mean(Std))**					
Total cholesterol	4.44(1.28)	3.98(1.12)	4.02(1.15)	4.23(1.34)	3.66(0.61)
LDL-cholesterol	2.46(0.90)	2.41(0.90)	2.44(0.90)	2.63(1.01)	2.11(0.56)
HDL-cholesterol	1.21(0.32)	1.21(0.36)	1.21(0.36)	1.23(0.30)	1.19(0.46)
Triglyceride	1.80(1.37)	1.56(0.93)	1.59(0.98)	1.73(1.12)	1.34(0.65)
**hs-C-reactive protein (mg/L)**	2.75(3.64)	2.07(3.62)	2.12(3.89)	2.43(4.52)	1.60(2.51)

Supplementary Table S2 Clinical characteristics of three subgroups in the Danhong injection group

Characteristics	D(+)S(+)	D(-)S(-)	D(-)S(+)
**Cluster**	Cluster 1	Cluster 2	Cluster 3
**Number**	122	162	298
**Sex —No. (%)**			
Male	86	102	195
Female	36	60	103
**Age (yr) (Mean(Std))**	60.40(6.80)	59.41(7.07)	58.89(6.79)
**BMI(kg/m^2) (Mean(Std))**	24.64(2.64)	24.34(2.78)	24.89(3.14)
**SAQ domains (Mean(Std))**			
Physical limitation	65.88(12.87)	68.66(12.82)	66.99(12.10)
Angina frequency	31.72(15.25)	68.33(21.13)	60.00(17.56)

Characteristics	D(+)S(+)	D(-)S(-)	D(-)S(+)
Treatment satisfaction	41.71(19.56)	59.59(18.36)	51.93(16.90)
Quality of life	32.58(16.39)	44.44(18.62)	37.34(18.16)
Angina stabilit	42.21(20.60)	46.30(24.00)	42.03(20.14)
**TCM syndrome score (Mean(Std))**	24.61(7.09)	20.56(9.15)	20.84(7.40)
**CCS angina class—No. (%)§**			
II	84	137	240
III	38	25	58
**Plasma lipid level (mmol/L)(Mean(Std))**			
Total cholesterol	4.31(1.13)	4.46(1.89)	4.46(1.04)
LDL-cholesterol	2.47(0.96)	2.50(0.92)	2.43(0.83)
HDL-cholesterol	1.14(0.32)	1.23(0.30)	1.24(0.29)
Triglyceride	1.95(1.50)	1.59(0.84)	1.83(1.68)
**hs-C-reactive protein (mg/L)**	3.34(4.88)	2.66(3.20)	2.68(2.86)

Supplementary Table S3: Detailed information of the 55 regulatory genes

		a				c			
**No.**	**gene**	**Estimate**	**Std. Error**	**Pr(>|t|)**	**p.adj**	**Estimate**	**Std. Error**	**Pr(>|t|)**	**p.adj**
1	CRK	-94.31135391	20.81809657	0.000137134	0.019589776	-0.044895866	0.01461255	0.005221479	0.019589776
2	GREB1	-1018.061331	262.7741107	0.000723504	0.025994853	-0.404226262	0.136576347	0.0068278	0.025994853
3	ARMCX2	-335.4827259	87.67378933	0.000815916	0.122406847	-0.326231164	0.085851655	0.000872174	0.006977389
4	CDH23	-78.0164963	20.66500791	0.000927809	0.020435741	-0.046574596	0.01703473	0.011564163	0.023128327
5	TMEM180	-203.1772532	54.83983199	0.001106605	0.020198768	-0.095910459	0.035744331	0.012996177	0.025992354
6	IQCD	57.40009	16.4464	0.00189	0.01510307	-0.675243669	0.313764841	0.041663694	0.11110318
7	BCAS1	-432.0878963	126.352314	0.002246046	0.12971274	-0.234342477	0.100365421	0.028241771	0.11296708
8	ELL3	-329.7664905	97.48800265	0.002460326	0.09170254	-0.274889365	0.090056856	0.005477488	0.02815777
9	PPP1R21	-92.68298415	27.60258032	0.002615095	0.090760732	-0.04489986	0.020760881	0.040746756	0.090760732
10	KIAA0391	3.732059	1.1494	0.00343	0.01370876	-0.056002015	0.022820663	0.021764875	0.058039667
11	JPX	-241.7870453	74.50892749	0.003442936	0.061512405	-0.139892926	0.056786947	0.021314097	0.061512405
12	FOS	-0.684868772	0.219056949	0.004586707	0.11500912	-0.000678478	0.000230247	0.007040466	0.05632372

13	PSMD10	-59.29271031	19.36498331	0.005355131	0.10436	-0.062840194	0.018886159	0.002817487	0.02253989
14	TRPC2	-418.5997912	137.0577344	0.005454145	0.06763149	-0.358644705	0.139197672	0.016559188	0.05508189
15	PDIA5	16.54238	5.47096	0.00587	0.02346386	-0.180586858	0.068201176	0.014088897	0.03757039
16	ARL2BP	1.61962	0.54119	0.00631	0.03097404	-0.027315296	0.012113432	0.033532281	0.08941942
17	SSBP2	-193.254141	66.14747371	0.007471245	0.06928237	-0.182112256	0.061966587	0.007172334	0.02868934
18	UQCRQ	-81.78031876	29.09170946	0.009675819	0.08490126	-0.047408508	0.021953716	0.041023822	0.10939686
19	MEGF11	3078.746765	1095.507151	0.00969343	0.1121277	2.172328304	0.933515958	0.028725507	0.1121277
20	HSBP1L1	5.45435	1.95986	0.01033	0.04131117	-0.07782991	0.033913119	0.030779926	0.0820798
21	SLC16A1	9.78543	3.55157	0.011	0.05145544	-0.188984293	0.086188473	0.038261981	0.10203195
22	PRRT1	27.87999	10.21587	0.0117	0.05417653	-0.355801525	0.15710365	0.03283852	0.06567704
23	SYS1	5.01864	1.87716	0.0133	0.08492494	-0.070909678	0.028763923	0.021231235	0.08492494
24	RACGAP1	9.69336	3.65565	0.014	0.05489968	-0.174584545	0.069700822	0.019446497	0.05489968
25	KCNG2	870.5230631	330.5283527	0.014548718	0.04777238	0.970039098	0.308257767	0.004366854	0.03493483
26	LDOC1	-125.5844307	47.68449445	0.014551313	0.0939954	-0.115364996	0.038451683	0.006201923	0.04961538
27	ATP6V1E2	-287.4308027	109.4576025	0.014807856	0.01819254	-0.28303264	0.090900091	0.004729743	0.01891897
28	SLC7A8	-359.9622492	138.486197	0.015730554	0.1359501	-0.371242099	0.150787751	0.021382691	0.1359501
29	IL23R	-730.5463562	281.7414992	0.015955964	0.1052483	-0.562558989	0.237617321	0.026312065	0.1052483
30	MLEC	-72.44437287	27.95555079	0.016012048	0.19198704	-0.070918078	0.025181492	0.009560427	0.07648342
31	TTC4	-110.3288709	43.49772607	0.018119045	0.13168877	-0.104363157	0.036174691	0.008142428	0.06513942
32	NHEJ1	-100.865066	41.0986295	0.021754541	0.02970402	-0.098437944	0.035471034	0.010517843	0.02970402
33	NOA1	1.9935	0.81998	0.02289	0.09155992	-0.041140842	0.018349538	0.034465707	0.09190855
34	LNX	-321.18	132.51	0.02326	0.0970	-0.31934	0.11519	0.01059	0.0437

	2	66168	17061	118	6963	1408	991	3623	68
35	S1PR3	2.861111	1.187197	0.024	0.09592069	-0.041700419	0.019392733	0.041816864	0.11151164
36	DPY30	-47.69312803	20.03611907	0.025585004	0.1178304	-0.04518187	0.019358872	0.028301867	0.1132075
37	MTCH2	3.51006	1.48234	0.02629	0.07010306	-0.082642438	0.033902715	0.02256537	0.07010306
38	DPAGT1	2.35282	0.99834	0.026936	0.071828837	-0.049919887	0.020539499	0.022926058	0.071828837
39	MB21D2	-448.7059863	191.6467928	0.02785158	0.1756181	-0.48757336	0.182094427	0.013165171	0.1053214
40	SFMBT1	6.3842	2.75549	0.02936	0.09703615	-0.136140665	0.061422284	0.036388556	0.09703615
41	CAMSAP2	20.84849	9.03327	0.02993	0.11366076	-0.328047514	0.153210335	0.042622787	0.11366076
42	TCEAL4	3.10972	1.36506	0.03192	0.08510785	-0.05393586	0.022026562	0.022022642	0.08510785
43	PEX2	8.73187	3.89238	0.03437	0.09166306	-0.188248645	0.078629058	0.024826425	0.09166306
44	ABCF2	2.32691	1.04337	0.0353	0.09760668	-0.058700111	0.026516757	0.036602507	0.09760668
45	CCR2	0.43886	0.201019	0.039	0.1281712	-0.005816539	0.002772326	0.04660671	0.1281712
46	ZFYVE9	-455.1512763	212.1197095	0.042218657	0.1310416	-0.444030661	0.214264004	0.049140583	0.1310416
47	ALG2	3.385009	1.590836	0.0438	0.1168595	-0.072652293	0.030209045	0.024243751	0.1168595
48	PDXDC1	-51.39264837	24.34932363	0.045411486	0.08750237	-0.055345809	0.021672747	0.01743067	0.08394706
49	MTFR1	-168.0146062	79.71027351	0.045677579	0.001221894	-0.190840253	0.067985486	0.009767316	0.026046176
50	SH3PXD2A	-212.8894523	101.1556665	0.045984982	0.08141413	-0.220877915	0.082147169	0.012831523	0.08141413
51	TMEM39B	-70.97699339	33.75158311	0.046142003	0.12579514	-0.067986065	0.026723492	0.017812064	0.07124826
52	PARP2	5.01837	2.39714	0.04705	0.11839888	-0.098334473	0.045504278	0.040893709	0.11839888
53	CTSG	-2.44779	1.17538	0.0481	0.1924831	0.054308221	0.022116011	0.021687975	0.1735038
54	ORM1	43.36227996	21.00784905	0.049979472	0.138064	0.041029799	0.017910353	0.031054576	0.138064
55	CTR	-498.27	197.27	0.01855	0.0539	-0.33368	0.15565	0.04239	0.0847

C	05021	20779	486344 38481	6442	8837	8224	5138	9028
