# Supplementary figures and images for: A Phenome-Wide Comparative Analysis of Individualized Network Heterogeneity Across Treatment-Response Subphenotypes in Coronary Heart Disease
Source: Biology (Basel). 2026 May 28;15(11):843. doi: 10.3390/biology15110843 (PMC13255898; doi:10.3390/biology15110843)

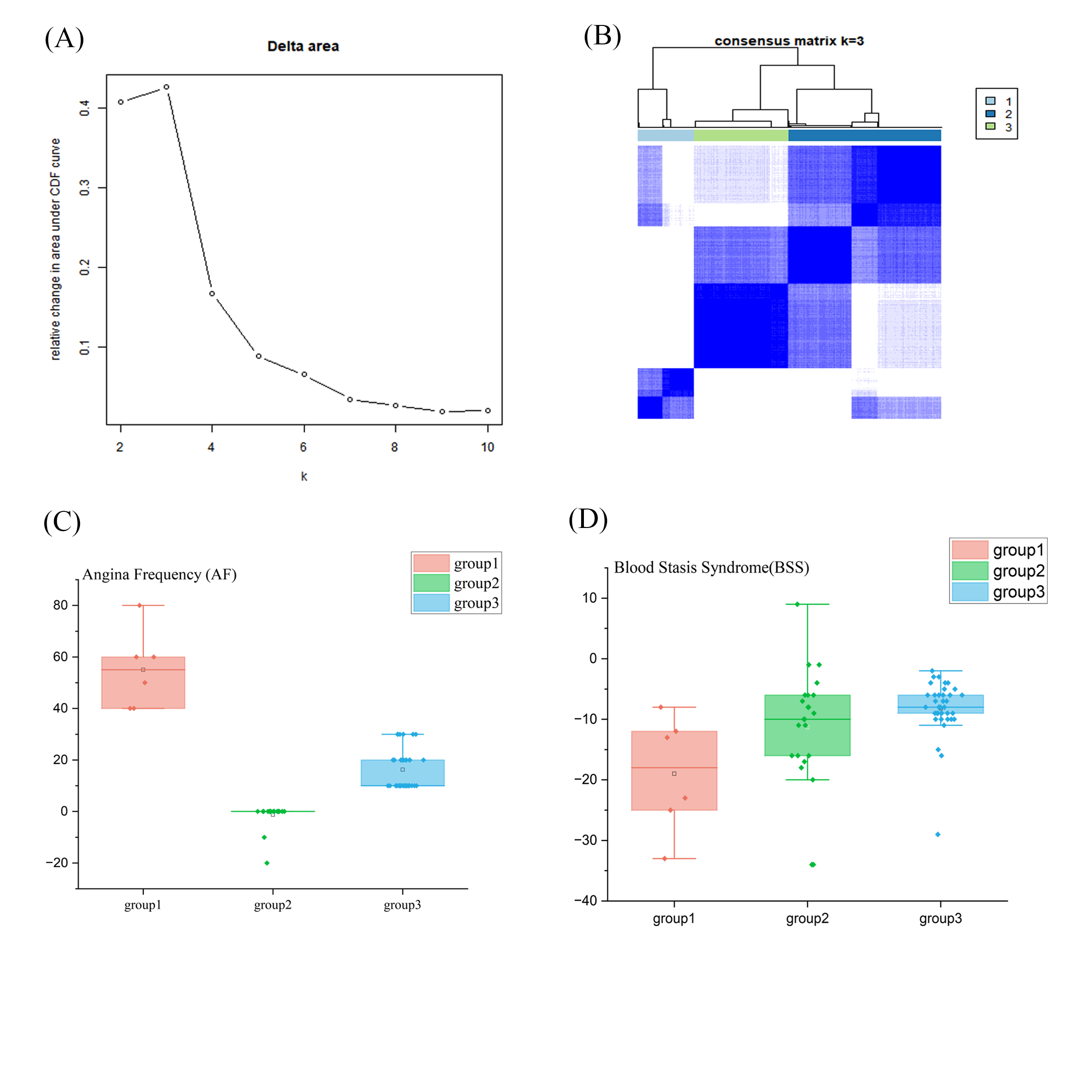

Supplement: Supplementary file 1 [file biology-15-00843-s001.zip › Supplementary Figure S1.tif]
